# Possible association between *Interleukin-1beta *gene and schizophrenia in a Japanese population

**DOI:** 10.1186/1744-9081-7-35

**Published:** 2011-08-16

**Authors:** Daimei Sasayama, Hiroaki Hori, Toshiya Teraishi, Kotaro Hattori, Miho Ota, Yoshimi Iijima, Masahiko Tatsumi, Teruhiko Higuchi, Naoji Amano, Hiroshi Kunugi

**Affiliations:** 1 Department of Mental Disorder Research, National Institute of Neuroscience, National Center of Neurology and Psychiatry, Kodaira, Tokyo, 187-8502, Japan; 2 Department of Psychiatry, Shinshu University School of Medicine, Matsumoto, 390-8621, Japan; 3 Core Research of Evolutional Science & Technology (CREST), Japan Science and Technology Agency (JST), Tokyo, 102-0075, Japan; 4 Department of Medical Genetics, Majors of Medical Sciences, Graduate School of Comprehensive Human Sciences, University of Tsukuba, Tsukuba, 305-8577, Japan; 5 Yokohama Shinryo Clinic, Yokohama, 221-0835, Japan; 6 National Center of Neurology and Psychiatry, Kodaira, Tokyo, 187-8502, Japan

## Abstract

**Background:**

Several lines of evidence have implicated the pro-inflammatory cytokine interleukin-1beta (IL-1β) in the etiology of schizophrenia. Although a number of genetic association studies have been reported, very few have systematically examined gene-wide tagging polymorphisms.

**Methods:**

A total of 533 patients with schizophrenia (302 males: mean age ± standard deviation 43.4 ± 13.0 years; 233 females; mean age 44.8 ± 15.3 years) and 1136 healthy controls (388 males: mean age 44.6 ± 17.3 years; 748 females; 46.3 ± 15.6 years) were recruited for this study. All subjects were biologically unrelated Japanese individuals. Five tagging polymorphisms of *IL-1β *gene (rs2853550, rs1143634, rs1143633, rs1143630, rs16944) were examined for association with schizophrenia.

**Results:**

Significant difference in allele distribution was found between patients with schizophrenia and controls for rs1143633 (*P *= 0.0089). When the analysis was performed separately in each gender, significant difference between patients and controls in allele distribution of rs1143633 was observed in females (*P *= 0.0073). A trend towards association was also found between rs16944 and female patients with schizophrenia (*P *= 0.032).

**Conclusions:**

The present study shows the first evidence that the *IL-1β *gene polymorphism rs1143633 is associated with schizophrenia susceptibility in a Japanese population. The results suggest the possibility that the influence of *IL-1β *gene variations on susceptibility to schizophrenia may be greater in females than in males. Findings of the present study provide further support for the role of IL-1β in the etiology of schizophrenia.

## Background

Several lines of evidence suggest that pro-inflammatory cytokine interleukin-1beta (IL-1β) is implicated in the etiology and pathophysiology of schizophrenia. Although studies investigating peripheral levels of IL-1β in schizophrenic patients have reported inconsistent results [1-6], a study examining the cerebrospinal fluid has shown a marked elevation of IL-1β in patients with first-episode schizophrenia compared to healthy controls [7]. Kowalski et al [8] reported that the release of IL-1β by peripheral monocytes was increased before treatment and then normalized by antipsychotic medication in patients with schizophrenia. Recently, Liu et al. [9] showed that IL-1β in the peripheral blood mononuclear cells was overexpressed not only in schizophrenia patients but also in their siblings, suggesting the involvement of the hereditary factors. Furthermore, previous findings suggested that IL-1β may be involved in the possible link between prenatal exposure to infection and schizophrenia [10,11].

The *IL-1β *gene is located in a region on 2q14. This region has consistently shown positive linkage findings in schizophrenia. Many studies have reported this region among their largest results [12,13]. Furthermore, Lewis et al [14] have shown in their meta-analysis of 20 genome scans that 2p12-q22.1 was associated with a genomewide significant *P *value. Linkage of this region with schizophrenia in an Asian population has also been reported [15].

A number of genetic association studies have suggested that genetic variation of the *IL-1β *gene might confer susceptibility to schizophrenia. Three studies in Caucasian populations reported a significant association of schizophrenia with an *IL-1β *gene polymorphism rs16944 [16-18]. However, this association was not confirmed in other studies [19,20]. Furthermore, none of the previous studies in Asian populations have obtained evidence for an association between *IL-1β *gene and schizophrenia [21-23]. All of the aforementioned association studies, except for that of Shirts, et al. [19], examined only rs16944 and/or rs1143634. Therefore, the role of other *IL-1β *gene polymorphisms remains to be determined. We here examined 5 tagging polymorphisms of the *IL-1β *gene for an association with schizophrenia in a Japanese sample.

## Methods

### Subjects

Subjects were 533 patients with schizophrenia (302 males: mean age ± standard deviation 43.4 ± 13.0 years; 233 females; mean age 44.8 ± 15.3 years) and 1136 healthy controls (388 males: mean age 44.6 ± 17.3 years; 748 females; 46.3 ± 15.6 years). The mean age at onset was 23.9 ± 8.0 and 25.8 ± 9.8 years for male and female patients, respectively. All subjects were biologically unrelated Japanese individuals, based on their self-reports, and were recruited from the outpatient clinic of the National Center of Neurology and Psychiatry Hospital, Tokyo, Japan or through advertisements in free local information magazines and by our website announcement. Consensus diagnosis by at least two psychiatrists was made for each patient according to the Diagnostic and Statistical Manual of Mental Disorders, 4^th ^edition criteria [24], on the basis of unstructured interviews and information from medical records. The controls were healthy volunteers with no current or past history of psychiatric treatment, and were screened using the Japanese version of the Mini International Neuropsychiatric Interview (M.I.N.I.) [25,26] by a research psychiatrist to rule out any axis I psychiatric disorders. Participants were excluded if they had prior medical histories of central nervous system disease or severe head injury, or if they met the criteria for substance abuse or dependence, or mental retardation. The study protocol was approved by the ethics committee at the National Center of Neurology and Psychiatry, Japan. After description of the study, written informed consent was obtained from every subject. Most of the subjects had participated in our previous genetic association studies [27,28]. Some of the control subjects had also participated in our previous studies which examined *IL-1β *gene polymorphisms [29,30].

### Genotyping

Five tagging single nucleotide polymorphisms (SNPs) (rs2853550, rs1143634, rs1143633, rs1143630, rs16944) in a region 1 kilobase (kb) upstream to 1 kb downstream of the *IL-1β *gene (chromosome 2: 113,302,808 - 113,311,827 bp) were selected by Haploview 4.2 [31] using Japanese and Chinese population in the HapMap SNP set (version 22), at an r^2 ^threshold of 0.80 with a minor allele frequency greater than 0.1. Genomic DNA was prepared from the venous blood according to standard procedures. The SNPs were genotyped using the TaqMan 5'-exonuclease allelic discrimination assay. Thermal cycling conditions for polymerase chain reaction were 1 cycle at 95°C for 10 minutes followed by 50 cycles of 92°C for 15 seconds and 60°C for 1 minute. The allele-specific fluorescence was measured with ABI PRISM 7900 Sequence Detection Systems (Applied Biosystems, Foster city, CA, USA). Genotype data were read blind to the case-control status. Ambiguous genotype data were not included in the analysis. The call rates for each SNP ranged from 97.7% to 98.6%. The genotyping failure rate for all SNPs combined was < 2%. In 92 subjects, all 5 SNPs were genotyped in duplicate to ensure genotyping accuracy, and the concordance rate of called genotypes was over 99%.

### Statistical analysis

Deviations of genotype distributions from the Hardy-Weinberg equilibrium (HWE) were assessed with the exact test described by Wigginton et al [32]. Genotype and allele distributions were compared between patients and controls by using the χ^2 ^test for independence or with Fisher's exact test. The above statistical analyses were performed using PLINK version 1.07 [33].

Haploview 4.2 [31] was used to estimate haplotype frequencies and linkage disequilibrium (LD) coefficients. Haplotypes with frequencies > 1% were included in the association analysis. Permutation procedure (10,000 replications) was used to determine the empirical significance.

Statistical tests were two tailed and statistical significance was considered when *P *< 0.05. Significance level corrected for multiple comparisons of 5 SNPs was set at *P *< 0.013 by a method proposed by Li et al [34], which was calculated using SNPSpD (SNP Spectral Decomposition) software [35].

Power calculations were performed using the Power Calculator for Two Stage Association Studies (http://www.sph.umich.edu/csg/abecasis/CaTS/). Power was calculated under prevalence of 0.01 using an allelic model with an alpha level of 0.05. Assuming disease allele frequencies of 0.20 and 0.40, our sample had 80% statistical power to detect relative risks of 1.28 and 1.23, respectively. Similarly, we had 90% power to detect relative risks of 1.33 and 1.27.

Since several aspects of immunity have marked sex differences [36], analyses were performed not only for the entire sample but also for each gender separately. Assuming allele frequency of 0.40, male and female samples each had 80% statistical power to detect relative risks of 1.35 and 1.34, respectively.

## Results

Genotype and allele distributions of the examined SNPs for the entire sample, males, and females are shown in Table 1, 2, and 3, respectively. The genotype distributions did not significantly deviate from the HWE in any of the SNPs examined. Significant differences in genotype and allele distributions were found between the patients with schizophrenia and controls for rs1143633. The C allele was significantly more common in patients than in controls (odds ratio 1.22, 95% confidence interval (CI) 1.05 to 1.41, *P *= 0.0089). This association remained significant after correcting for multiple testing of 5 SNPs (corrected *P *= 0.013). When the analysis was performed separately in each gender, significant difference between patients and controls in allele distribution of rs1143633 was observed only in females (odds ratio 1.34, 95% CI 1.08 to 1.66, *P *= 0.0073). The A allele of rs16944 also showed a trend towards association with schizophrenia in female subjects (odds ratio 1.26, 95% CI 1.02 to 1.56, *P *= 0.032).

**Table 1:** Association analysis of the 5 SNPs in both genders combined

			Males								
			
SNP name	Allele 1/2		N	Genotype			Allele		P-value		HWE P-value
							
				1/1	1/2	2/2	1	2	Genotype	Allele	
rs2853550	A/G	Schizophrenia	531	9	128	394	146	916	0.23	0.088	0.86
				(0.02)	(0.24)	(0.74)	(0.14)	(0.86)			
		Controls	1115	14	232	869	260	1970			0.88
				(0.01)	(0.21)	(0.78)	(0.12)	(0.88)			
rs1143634	A/G	Schizophrenia	525	1	41	483	43	1007	0.97^(a)^	0.90	0.59
				(0.00)	(0.08)	(0.92)	(0.04)	(0.96)			
		Controls	1121	2	90	1029	94	2148	1.00		
				(0.00)	(0.08)	(0.92)	(0.04)	(0.96)			
rs1143633	C/T	Schizophrenia	524	111	249	164	471	577	0.035	0.0089	0.38
				(0.21)	(0.48)	(0.31)	(0.45)	(0.55)			
		Controls	1123	188	525	410	901	1345			0.38
				(0.17)	(0.47)	(0.37)	(0.40)	(0.60)			
rs1143630	T/G	Schizophrenia	520	13	140	367	166	874	0.88	0.66	1.00
				(0.03)	(0.27)	(0.71)	(0.16)	(0.84)			
		Controls	1119	24	296	799	344	1894			0.65
				(0.02)	(0.26)	(0.71)	(0.15)	(0.85)			
rs16944	A/G	Schizophrenia	521	123	253	145	499	543	0.18	0.060	0.54
				(0.24)	(0.49)	(0.28)	(0.48)	(0.52)			
		Controls	1111	226	534	351	986	1236			0.39
				(0.20)	(0.48)	(0.32)	(0.44)	(0.56)			

(a) Calculated using Fisher's exact test.

SNP: single nucleotide polymorphism; HWE: Hardy-Weinberg Disequilibrium

Numbers in parentheses represent the frequencies of genotypes and alleles.

**Table 2:** Association analysis of the 5 SNPs in males

			Males								
			
SNP name	Allele 1/2		N	Genotype			Allele		P-value		HWE P-value
							
				1/1	1/2	2/2	1	2	Genotype	Allele	
rs2853550	A/G	Schizophrenia	300	4	74	222	82	518	0.68^(a)^	0.69	0.62
				(0.01)	(0.25)	(0.74)	(0.14)	(0.86)			
		Controls	383	7	85	291	99	667			0.82
				(0.02)	(0.22)	(0.76)	(0.13)	(0.87)			
rs1143634	A/G	Schizophrenia	298	0	24	274	24	572	0.81^(a)^	0.82	1.00
				(0.00)	(0.08)	(0.92)	(0.04)	(0.96)			
		Controls	383	1	27	355	29	737			0.42
				(0.00)	(0.07)	(0.93)	(0.04)	(0.96)			
rs1143633	C/T	Schizophrenia	299	59	145	95	263	335	0.43	0.47	0.81
				(0.20)	(0.48)	(0.32)	(0.44)	(0.56)			
		Controls	383	77	168	138	322	444			0.059
				(0.20)	(0.44)	(0.36)	(0.42)	(0.58)			
rs1143630	T/G	Schizophrenia	295	7	81	207	95	495	0.75	0.73	1.00
				(0.02)	(0.27)	(0.70)	(0.16)	(0.84)			
		Controls	383	6	106	271	118	648			0.32
				(0.02)	(0.28)	(0.71)	(0.15)	(0.85)			
rs16944	A/G	Schizophrenia	295	66	143	86	275	315	0.92	0.67	0.64
				(0.22)	(0.48)	(0.29)	(0.47)	(0.53)			
		Controls	385	82	186	117	350	420			0.61
				(0.21)	(0.48)	(0.30)	(0.45)	(0.55)			

(a) Calculated using Fisher's exact test.

SNP: single nucleotide polymorphism; HWE: Hardy-Weinberg Disequilibrium

Numbers in parentheses represent the frequencies of genotypes and alleles.

**Table 3:** Association analysis of the 5 SNPs in females

			Males								
			
SNP name	Allele 1/2		N	Genotype			Allele		P-value		HWE P-value
							
				1/1	1/2	2/2	1	2	Genotype	Allele	
rs2853550	A/G	Schizophrenia	231	5	54	172	64	398	0.18	0.096	0.78
				(0.02)	(0.23)	(0.74)	(0.14)	(0.86)			
		Controls	732		7	147	578		161	1303	0.57
				(0.01)	(0.20)	(0.79)	(0.11)	(0.89)			
rs1143634	A/G	Schizophrenia	227	1	17	209	19	435	0.46^(a)^	0.84	0.32
				(0.00)	(0.07)	(0.92)	(0.04)	(0.96)			
		Controls	738	1	63	674	65	1411			1.00
			(0.00)	(0.09)	(0.91)	(0.04)	(0.96)				
rs1143633	C/T	Schizophrenia	225	52	104	69	208	242	0.013	**0.0073**	0.29
			(0.23)	(0.46)	(0.31)	(0.46)	(0.54)				
		Controls	740	111	357	272	579	901			0.76
			(0.15)	(0.48)	(0.37)	(0.39)	(0.61)				
rs1143630	T/G	Schizophrenia	225	6	59	160	71	379	0.97	0.83	0.80
			(0.03)	(0.26)	(0.71)	(0.16)	(0.84)				
		Controls	736	18	190	528	226	1246			0.89
			(0.02)	(0.26)	(0.72)	(0.15)	(0.85)				
rs16944	A/G	Schizophrenia	226	57	110	59	224	228	0.11	0.032	0.69
			(0.25)	(0.49)	(0.26)	(0.50)	(0.50)				
		Controls	726	144	348	234	636	816			0.50
			(0.20)	(0.48)	(0.32)	(0.44)	(0.56)				

(a) Calculated using Fisher's exact test.

SNP: single nucleotide polymorphism; HWE: Hardy-Weinberg Disequilibrium

Numbers in parentheses represent the frequencies of genotypes and alleles. Significant P-values (< 0.013) are shown in boldface.

**Figure 1:**
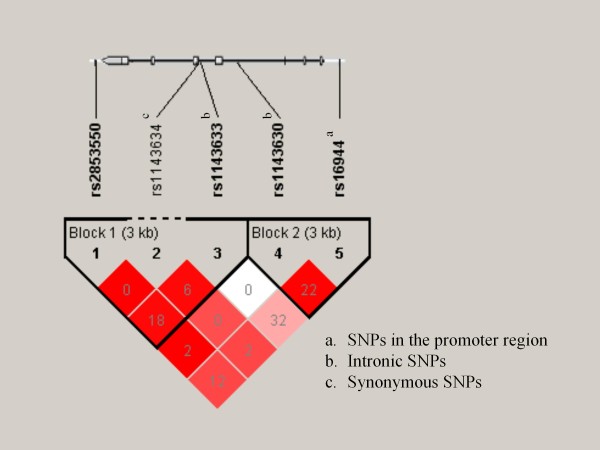
**Haplotype block structure of the *IL-1β *gene**. Genomic organization and linkage disequilibrium (LD) structure of the *IL-1β *gene are shown. Exons are shown as boxes. Shades of red represent extent of LD (darker red denotes D' = 1). Numbers in squares give r^2 ^values multiplied by 100.

Linkage disequilibrium (LD) coefficients (D' and r^2^) and haplotype blocks are shown in Figure 1. Results of the haplotype association analyses are shown in Table 4. No significant difference in haplotype distribution was found between patients with schizophrenia and controls (all *P *> 0.05 by permutation test).

**Table 4:** Haplotype analysis of *IL-1β *gene polymorphisms

			Males					Females				
			
Block	Haplotype	Diagnosis	Carrier	Non-carrier	χ2	Nominal P value	Permutation P value	Carrier	Non-carrier	χ2	Nominal P value	Permutation P value
	GT	Schizophrenia	336.3	265.7	0.557	0.456	0.957	251.0	213.0	6.240	0.0125	0.118
			(0.559)	(0.441)				(0.541)	(0.459)			
		Controls	447.9	326.1				901.0	585.0			
			(0.579)	(0.421)				(0.606)	(0.394)			
1	GC	Schizophrenia	183.1	418.9	0.216	0.642	0.995	149.0	315.0	2.298	0.130	0.691
			(0.304)	(0.696)				(0.321)	(0.679)			
		Controls	226.4	547.6				422.5	1063.5			
			(0.293)	(0.707)				(0.284)	(0.716)			
	AC	Schizophrenia	82.6	519.4	0.215	0.643	0.995	63.7	400.3	3.281	0.0701	0.461
			(0.137)	(0.863)				(0.137)	(0.863)			
		Controls	99.6	674.4				158.5	1327.5			
			(0.129)	(0.871)				(0.107)	(0.893)			
	GG	Schizophrenia	321.4	280.6	0.154	0.694	0.996	231.2	228.8	5.012	0.0252	0.207
			(0.534)	(0.466)				(0.503)	(0.497)			
		Controls	422.6	353.4				837.4	652.6			
			(0.545)	(0.455)				(0.562)	(0.438)			
2	GA	Schizophrenia	183.5	418.5	0.040	0.841	1.00	156.4	303.6	5.326	0.0210	0.178
			(0.305)	(0.695)				(0.340)	(0.660)			
		Controls	232.7	543.3				422.8	1067.2			
			(0.300)	(0.700)				(0.284)	(0.716)			
	TA	Schizophrenia	97.1	504.9	0.081	0.776	0.999	72.4	387.6	0.027	0.869	1.00
			(0.161)	(0.839)				(0.157)	(0.843)			
		Controls	120.7	655.3				229.8	1260.2			
			(0.156)	(0.844)				(0.154)	(0.846)			

Numbers in parentheses represent the frequencies of haplotypes. Permutation P values were based on 10,000 permutations.

## Discussion

To our knowledge, the present study is the largest study to date that examined the *IL-1β *gene polymorphisms for association with schizophrenia. The results provide the first evidence suggesting that the C allele of rs1143633 is associated with schizophrenia.

The study in a United States population by Shirts et al [19] was the only one that previously examined the association of schizophrenia with rs1143633, in which no significant difference was found in allele frequencies between patients and controls. Although Watanabe et al [23] have also examined 9 SNPs of the IL-1 gene complex in Japanese subjects, none of the SNPs examined in their study was in remarkable linkage disequilibrium with rs1143633 or rs16944 (all r^2 ^< 0.1 based on HapMap Japanese and Han Chinese population data, release 22). The inconsistent results regarding the effect of rs1143633 between Shirts, et al [19] and our study may be attributable to ethnic difference. Indeed, a recent meta-analysis has shown a significant association of the G allele of rs16944 and the G allele carrier status of rs1143634 with a risk of schizophrenia in Caucasian, but not in Asian, populations [37]. Our samples provided sufficient power to detect relatively small relative risks, and therefore suggest that rs16944 and rs1143634 have no major effect on schizophrenia susceptibility in Asian populations, which is consistent with the previous Asian findings [21-23]. However, there was a trend of association of rs16944, in the opposite direction to that of the Caucasians, with schizophrenia susceptibility in female subjects. Therefore, there remains a possibility that a larger study would yield a significant difference between Japanese female schizophrenic patients and controls in the allele frequency of rs16944.

A number of genome-wide association studies (GWAS) have searched for polymorphisms associated with schizophrenia [38-43]. Although no evidence of association with *IL-1β *gene has been reported, common risk alleles in the major histocompatibility region on chromosome 6, which is involved in the immune response, have shown statistically significant evidence of association [38-40]. Furthermore, a genome-wide pharmacogenomic study has shown that *IL-1α *rs11677416, which is in weak LD with rs1143633 (r^2 ^= 0.094, D' = 0.809 based on HapMap Japanese and Han Chinese population data, release 22), was associated with response of neurocognitive symptoms to antipsychotic treatment [44]. These findings, together with ours, suggest genetic influence on immune alterations in schizophrenia.

A shift towards the T helper type 2 (Th2) system has been indicated in schizophrenia [45-47]. IL-1β stimulates the production of prostaglandin E2, which is an important cofactor for the induction of T-helper lymphocyte activity towards Th2 direction. Significant increase in circulating mRNA expression levels of IL-1β has been observed in schizophrenic patients [9]. The changes in mRNA levels may reflect the genetic variation in *IL-1β *gene. The findings on biological roles of *IL-1β *polymorphisms, however, have not been consistent across studies. A/A genotype of rs16944 has been associated with higher gastric mucosa IL-1β levels in H. pylori positive population [48]. On the other hand, subjects with G/G genotype showed an increased release of IL-1β from mononuclear cells after stimulation with lipopolysaccharide [49]. Recent studies suggest that the functional role of rs16944 may depend on the *IL-1β *promoter region haplotypes including rs16944 and rs1143627 [50-53]. Although the findings are inconsistent, these previous studies suggest that rs16944 could affect the expression levels of IL-1β. On the other hand, the influence of rs1143633 on IL-1β expression levels has not been previously reported.

Intriguingly, rs1143633 and rs16944 have also been associated with cortisol response to dexamethasone in healthy subjects [30]. Alleles associated with increased cortisol response to dexamethasone were shown to be associated with schizophrenia in the present study. Higher rates of non-suppression to dexamethasone compared to healthy subjects have been reported in schizophrenia [54] and schizotypy [55]. On the other hand, Ismail et al [56] reported that less than 2% of their schizophrenic patients were non-suppressors. Although the findings are inconsistent, these studies indicate that schizophrenia may be associated with alteration in hypothalamic- pituitary- adrenal (HPA) axis. Taken together, our findings suggest that *IL-1β *gene polymorphisms may play a role in the HPA axis alteration in schizophrenic patients.

Our results showed significant association of rs1143633 with schizophrenia in only females. Although our male sample was not large enough to detect a small relative risk, our data suggest that susceptibility to schizophrenia is more influenced by the *IL-1β *gene variation in females. To our knowledge, no previous studies have examined the gender differences in the association between *IL-1β *gene polymorphisms and schizophrenia. However, gender differences have been reported in the association between schizophrenia and RELA gene [27] encoding the major component of NF-κB, which is activated by IL-1β. Taken together with our results, the influence of IL-1β on susceptibility to schizophrenia may differ between genders. Indeed, gender differences in immunity have been reported in previous studies [36]. IL-1 release from mononucleated cells has been shown to be menstrual phase dependent in females and lower in males [57]. Furthermore, in vitro stimulation of lymphocytes with phytohemagglutinin has shown that females produce more Th2 cytokines than males [58]. Thus, future studies investigating associations of immune-related genes with schizophrenia should take into consideration the possible gender differences.

There are some limitations to this study. The ethnicity of the participants was based on self-reports and was not confirmed by genetic analyses. Our positive results might be derived from sample bias due to population stratification, although the Japanese are a relatively homogeneous population. Furthermore, structured interview such as SCID (Structured Clinical Interview for DSM) was not used for diagnosis in this study. Finally, the function of the *IL-1β *gene SNPs are unclear. Future studies are necessary to elucidate the function and its relationship with the pathogenesis of schizophrenia.

## Conclusions

Our results suggest that rs1143633 of *IL-1β *gene is associated with schizophrenia susceptibility in a Japanese population and that the influence of *IL-1β *gene variations on susceptibility to schizophrenia may be greater in females than in males. We obtained no significant evidence for a well-studied polymorphism rs16944 being associated with schizophrenia, which is consistent with previous studies in Asian populations. However, a trend of higher A allele frequency of rs16944 in female patients with schizophrenia leaves open a possibility that a larger study may yield a significant difference. The results of the present study provide further support for the role of IL-1β in the etiology of schizophrenia. Future studies are warranted to replicate the present findings and to reveal the functional role of *IL-1β *gene in pathophysiology of schizophrenia.

## Competing interests

The authors declare that they have no competing interests.

## Authors' contributions

DS and HK designed the study and DS wrote the draft of the manuscript. DS, HH, TT, KH, MO, MT, and HK made the diagnosis according to DSM-IV criteria. DS, HH, TT, KH, MO, and HK screened the healthy participants using the Mini International Neuropsychiatric Interview (M.I.N.I.). DS and YI performed the genotyping. HK supervised the data analysis and writing of the paper. TH and NA also supervised the writing of the paper and gave critical comments on the manuscript. All authors contributed to and have approved the final manuscript.
